# Composition of the microbial community in surface flow-constructed wetlands for wastewater treatment

**DOI:** 10.3389/fmicb.2024.1421094

**Published:** 2024-07-19

**Authors:** Haider Ali, Yongen Min, Xiaofei Yu, Yahya Kooch, Phyoe Marnn, Sarfraz Ahmed

**Affiliations:** ^1^Engineering Research Center of Low-Carbon Treatment and Green Development of Polluted Water in Northeast China, Ministry of Education and State Environmental Protection Key Laboratory For Wetland Conservation and Vegetation Restoration, School of Environment, Northeast Normal University, Changchun, China; ^2^Key Laboratory of Vegetation Ecology of Ministry of Education and Key Laboratory of Geographical Processes and Ecological Security of Changbai Mountains, Ministry of Education, School of Geographical Sciences, Northeast Normal University, Changchun, China; ^3^Heilongjiang Xingkai Lake Wetland Ecosystem National Observation and Research Station and Key Laboratory of Wetland Ecology and Environment and Jilin Provincial Joint Key Laboratory of Changbai Mountain Wetland and Ecology, Northeast Institute of Geography and Agroecology, Chinese Academy of Sciences, Changchun, China; ^4^Faculty of Natural Resources and Marine Sciences, Tarbiat Modares University, Noor, Iran; ^5^School of Life Sciences, Northeast Normal University, Changchun, China; ^6^Key Laboratory of Remote Sensing, Northeast Institute of Geography and Agroecology, Chinese Academy of Sciences, Changchun, China

**Keywords:** microbial community, constructed wetland, lignite, crushed bricks, pollutant removal

## Abstract

Traditionally constructed wetlands face significant limitations in treating tailwater from wastewater treatment plants, especially those associated with sugar mills. However, the advent of novel modified surface flow constructed wetlands offer a promising solution. This study aimed to assess the microbial community composition and compare the efficiencies of contaminant removal across different treatment wetlands: CW1 (Brick rubble, lignite, and *Lemna minor* L.), CW2 (Brick rubble and lignite), and CW3 (*Lemna minor* L.). The study also examined the impact of substrate and vegetation on the wetland systems. For a hydraulic retention time of 7 days, CW1 successfully removed more pollutants than CW2 and CW3. CW1 demonstrated removal rates of 72.19% for biochemical oxygen demand (BOD), 74.82% for chemical oxygen demand (COD), 79.62% for NH_4_^+^-N, 77.84% for NO_3_^−^-N, 87.73% for ortho phosphorous (OP), 78% for total dissolved solids (TDS), 74.1% for total nitrogen (TN), 81.07% for total phosphorous (TP), and 72.90% for total suspended solids (TSS). Furthermore, high-throughput sequencing analysis of the 16S rRNA gene revealed that CW1 exhibited elevated Chao1, Shannon, and Simpson indices, with values of 1324.46, 8.8172, and 0.9941, respectively. The most common bacterial species in the wetland system were Proteobacteria, Spirochaetota, Bacteroidota, Desulfobacterota, and Chloroflexi. The denitrifying bacterial class Rhodobacteriaceae also had the highest content ratio within the wetland system. These results confirm that CW1 significantly improves the performance of water filtration. Therefore, this research provides valuable insights for wastewater treatment facilities aiming to incorporate surface flow-constructed wetland tailwater enhancement initiatives.

## 1 Introduction

Rapid urbanization and the continuous growth of industrial production and technology have led to severe water shortages and raised pollution in recent years. Maintaining water quality has become a critical global issue (Kadier et al., 2016; Lu et al., 2016; Qi et al., 2024). Discharging of untreated or partially treated industrial wastewater into local water bodies disrupts ecological sustainability and poses significant human health risks (Jacobson et al., 2013; Hasan and Webley, 2017; Swain et al., 2018). Excessive nitrogen and phosphorus emissions significantly impact the structure and function of river ecosystems, aggravating water eutrophication and disrupting microbial community structures (Huo et al., 2017). Sugar industries are among the most polluting industries (Ingaramo et al., 2009). Molasses, a byproduct of the sugarcane industry, is used as a raw material for ethyl alcohol production, generating 15 liters of spent wash for every liter of the alcohol produced. Inadequate treatment of this spent wash, which has a high organic load, biochemical oxygen demand (BOD) levels between 35,000 and 60,000 mg/L, and chemical oxygen demand (COD) levels between 60,000 and 120,000 mg/L, can have devastating effects on ecosystems (Billore et al., 2001; Zurita and Vymazal, 2023). Anaerobic biological digestion (biomethanation) is a commonly employed traditional treatment technology that effectively mitigates the elevated organic load in wastewater. Distilleries typically use biomethanation to produce methane, which serves as fuel to meet energy requirements. A secondary treatment is implemented, which requires ongoing aeration and substantial energy input. Conventional effluent treatment plants (ETPs) produce high-quality wastewater in the outfalls of the secondary treatment system. This secondary treatment effluent (STE) is usually transported to open earthen lagoons for further natural treatment and is often scattered across open fields for sun drying. This process requires significant land and poses a risk of contaminating groundwater (Billore et al., 2001). In contrast, constructed wetland water treatment technology is recognized for its ability to provide environment friendly solutions with high treatment efficiency at a low cost (Ameso et al., 2023). It is widely used in the treatment of domestic sewage (Lai et al., 2020), agricultural runoff sewage (Vymazal and Březinová, 2015), and industrial and municipal wastewater (Wu et al., 2014; Gupta et al., 2020).

Studies have found that physical structure and operational strategies strongly influence the pollutant removal performance of constructed wetlands (Lu et al., 2016). The efficacy of pollutant removal depends on aerobic, anaerobic, and other active conditions and the type of filler used. Different fillers support the growth of various microorganisms in the substrate layer, which play a vital role in the removal process (Hussain et al., 2019). Removing organic matter and ammonia nitrogen in constructed wetlands mainly depends on their adsorption to substrates and microbial degradation (Kizito et al., 2017). Duckweed-based ponds utilizing *Lemna minor* L. have effectively eliminated nutrients and organic substances (Papadopoulos and Tsihrintzis, 2011), combining proficient wastewater treatment with significant biomass generation (Iatrou et al., 2017). Under natural climatic conditions, duckweed demonstrated superior efficiency in removing chemical oxygen demand, nitrogen (N), and phosphorous (P) from dumpsite leachate (Iqbal et al., 2019). Crushed bricks have a positive impact on the development of plants and microorganisms. Hollow brick crumbs and fly ash are particularly effective in removing total nitrogen (Li H. et al., 2021) and total phosphorous (TP) (Li et al., 2022). Constructed wetlands containing a combination of hollow brick crumbs and fly ash can reduce NH_4_^+^-N levels by 89% and TP levels by 81% (Ren et al., 2007; Kumar and Dutta, 2019; Nan et al., 2020). Lignite, commonly known as brown coal, is renowned for its adsorptive characteristics. Numerous studies have used lignite as an adsorbent to eliminate heavy metals, organic contaminants, and colorants or dyes from wastewater (Allen et al., 1997; Mohan et al., 2002; Rathi and Puranik, 2002; Kluçáková and Omelka, 2004; Pehlivan and Arslan, 2006; Karaca et al., 2008; Havelcová et al., 2009; Pentari et al., 2009). Numerous comparison studies have examined the treatment performance of various substrates or vegetation in wetlands. Most of these studies have only reviewed the comparison between substrates or vegetation individually, without considering the combined impact of substrates and vegetation (Calheiros et al., 2009; Saeed and Sun, 2011, 2013; Sehar et al., 2015; Toscano et al., 2015; Schierano et al., 2017).

This research addresses the existing knowledge gap by examining the significance of substrate and vegetation in the context of constructed wetland applications. Prior studies have shown that *Lemna minor* L. can absorb pollutants from wastewater and is recognized as a hyperaccumulator for phosphorus. Additionally, porous media such as brick rubble and lignite not only adsorb toxins from wastewater but also create an environment for the growth of microbial communities (Allen et al., 1997; Mohan et al., 2002; Rathi and Puranik, 2002; Kluçáková and Omelka, 2004; Pehlivan and Arslan, 2006; Ren et al., 2007; Karaca et al., 2008; Havelcová et al., 2009; Pentari et al., 2009; Iqbal et al., 2019; Kumar and Dutta, 2019; Nan et al., 2020). Therefore, it is postulated that the implementation of a constructed wetland system that incorporates brick rubble and lignite (as substrates), along with *Lemna minor* L. (as a vegetation plant), may offer a viable, cost-effective, and environmentally sustainable approach to address the issue of wastewater from the effluent treatment plant for secondary treatment. This study was carried out to evaluate the efficiency of a small-scale surface flow-constructed wetland (SFCW) in treating secondary treatment effluent. The primary objectives were to examine the structure of the microbial community and evaluate the efficiency of constructed wetlands in removing organic compounds, specifically COD and BOD, as well as nitrogen and phosphorus. The ultimate goal was to enhance the quality of effluent from sugar industries and mitigate their negative impact on natural water systems, an urgent global problem.

## 2 Methodology

### 2.1 Experimental procedure

The whole experiment spans 120 days, from mid-May 2023 to mid-August 2023. The experiment consists of the vegetation period and the treatment phase. The vegetation period lasted for 90 days, during which the constructed wetlands were filled with tap water. The *Lemna minor* L. plants were distributed on the water surface to promote the development of the microbial community within the substrates. During the treatment phase, which started in mid-July, built wetlands were filled with synthetic sugar industrial effluent with hydraulic retention times (HRT) of 3, 6, and 7 days to determine optimal HRT throughout the treatment phase. The study assessed the performance of three different treatment configurations: substrate alone, vegetation alone, and substrate and vegetation combined. High-throughput sequencing of microbial community 16S rRNA was used to assess the impact and function of the microbial community in the treatment process. Additionally, the individual contributions of *Lemna minor* L. and Substrates (Brick rubble and lignite) to the treatment system were evaluated to determine their impact on the overall performance of the Surface Flow Constructed Wetland (SFCW).

### 2.2 Synthetic wastewater and wetland reactors

Synthetic wastewater (SWW) provides a controlled environment for thoroughly examining each parameter. With a few adjustments, a synthetic formulation of anaerobically treated distillery wastewater was created based on the specifications in Table 1 (Nacheva et al., 2009).

**Table 1:** Characteristics of wastewater (concentrations of all parameters are in mg/l.)

Parameter	Concentration
BOD	3,200
COD	7,500
NH_4_^+^-N	3.85
NO_3_^−^-N	20
OP	2.93
TDS	2,000
TN	35
TP	12
TSS	1,800

Synthetic sugarcane industry wastewater was developed in the laboratory by using a locally purchased sugarcane plant bagasse waste. It was then soaked in the water, and the containers were covered with a lid to prevent light penetration for 15 days to produce foul-smelling. This process made a real replicate of the effluent of sugar industry wastewater. Nitrogen (N) and Phosphorus (P) were added as NaNO_3_ (≥99%)_,_ HNO_3_ (≥99%), and NH_4_H_2_PO_4_ (≥98%), respectively. While NaOH (≥98%) and H_2_SO_4_ (≥98%) were used to control the pH. KCl (≥98%) was used for optimizing total suspended solids (TSS). The quantity of NaOH and H_2_SO_4_ was dependent on the buffer capacity of the water. The detailed composition of wastewater effluent is given in Table 1.

Rectangular-shaped wetlands were made of thick polythene plastic with dimensions of 50 cm*50 cm*60 cm. Three holes were drilled into the outer wall of each wetland, and ½ inches of valves were fixed in them, acting as an inlet (at the height of 200 mm), outlet (at 600 mm height), and drain (at 50 mm height) (Supplementary Figure S1).

Common clay bricks were purchased from the local brick industry and crushed to make brick rubble, which was used in combination with Lignite (Brown coal) as substrate. *Lemna Minor* L. was purchased from a local market and quickly transferred to wetlands reactors. The wetlands configuration consisted of T1 (CW1), *Lemna minor* L. (170 g) as vegetation plant, lignite (1,000 g), and Brick rubble (50 kg) as substrates; T2 (CW2), only substrates containing Brick Rubble (50 kg) and Lignite (1,000 g); T3 (CW3) with *Lemna minor* L. (170 g) only. All wetlands T1, T2, and T3 had three replicates and had a free water surface level of 40 cm on the top of the substrates in the tank.

### 2.3 Sample collection and measuring methods

#### 2.3.1 Influent and effluent water

Samples of influent and effluent water were taken from different systems to assess how well each system had removed the pollutants. Each time, 1 L of water was sampled using different hydraulic retention times to enable the examination of all indicators. The samples were obtained from the effluent valve of each wetland reactor during the treatment phase. They were then kept in a refrigerator at 4°C until all laboratory testing procedures were completed.

The pH and total dissolved solids (TDS) of the water samples were analyzed using a portable water quality meter (METTLER TOLEDO Co. Ltd., based in Switzerland and America). TN, NH_4_^+^-N, NO_3_^−^-N, COD, ortho phosphorous (OP), and TP concentrations were measured using a fully automated multipara meter and water quality analyzer (SMART CHEN 200, United States). The dissolved oxygen (DO) concentration was measured using a portable DO meter (HACH HQ30d, United States). Total suspended solids were determined using the APHA (American Public Health Association) standard procedure (American Public Health Association, 1926). All experimental data were presented as the mean of three replicates with standard deviation.

The parameters mentioned above were employed for assessing wastewater treatment efficacy, encompassing the quantification of removal efficiency (R, %), as specified by the subsequent equation.


$$R\%=\frac{\mathrm{Ci}-Cf}{\mathrm{Ci}}\times 100$$

The variable *R* represents the percentage removal efficiency, *Ci* represents the starting concentration of the influent, and *Cf* represents the final concentration of the effluent.

#### 2.3.2 Microbiology

The microbial samples were extracted from the substrate using 0.1 M phosphate buffer solution. Total genome DNA from samples was extracted using the CTAB/SDS method. The DNA concentration and purity were monitored on 1% agarose gel. It was diluted to 1 ng/μL using sterile water. The concentration was determined using the Qubit dsDNA HS Assay Kit and the Qubit 4.0 Fluorometer (Invitrogen, Thermo Fisher Scientific, Oregon, United States). The universal primer sets 341F: 5′-CCTAYGGGRBGCASCAG-3′ and 806R: 5′-GGACTACNNGGGTATCTAAT-3′ were used to amplify the V3-V4 region of the 16S rRNA gene from genomic DNA extracted from each sample. The sequencing was done on the Illumina NovaSeq platform by Biomarker Technologies Co., Ltd. The raw sequences obtained from sequencing were filtered using Trimmomatic (v0.33^1^). Primer sequences were removed using Cutadapt (v1.9.1^2^) to obtain clean sequences. The DADA2 (Callahan et al., 2016) method in QIIME2 2020.6 was used for denoising, bipartite sequence splicing, and removal of chimeric sequences to obtain the final valid data 346,434,000 bp. The number of base pairs used to develop the error model was 100,000,000 bp. Amplicon Sequence Variants (ASVs) were identified from high-quality sequences using Vsearch (v2.13.4_linux_x86_64). The effective sequences from sequencing are clustered based on 100% similarity. Using the classify-sklearn algorithm in QIIME2,^3^ a pre-trained Naive Bayes classifier was applied to annotate the species for each ASV. The annotation database used was Silva 138.1.^4^

### 2.4 Analysis

Statistical analyses were conducted using SPSS 27.0 (SPSS, Chicago, United States) to evaluate and compare the performance of various wetland reactor treatments. Prior to analysis, the Shapiro–Wilk test was applied to all data to assess normality, and Bartlett’s test was used to examine the homogeneity of variances. Depending on the distribution characteristics of the estimated parameters, either ANOVA or the Kruskal-Wallis test was employed for significant difference analysis among multiple groups. Pairwise data comparisons were conducted using Tukey’s HSD test or the Wilcoxon rank-sum test. All experimental data were presented as the mean with standard deviation. Statistical significance was determined at a *p*-value of less than 0.05.

Microbial community structure was analyzed through Principal Coordinates Analysis (PCoA) based on the Bray-Curtis distance matrix, with significance testing (PERMANOVA test, Adonis tool) performed using 999 permutations, facilitated by the “vegan” package of R (R Core Team, Vienna, Austria). Spearman correlation tests were used to determine the relationships between microbial community and pollution removal using the “psych” package of R (R Core Team, Vienna, Austria).

## 3 Results

### 3.1 Total nitrogen, NH_4_^+^-N and NO_3_^−^-N removal

**Figure 1:**
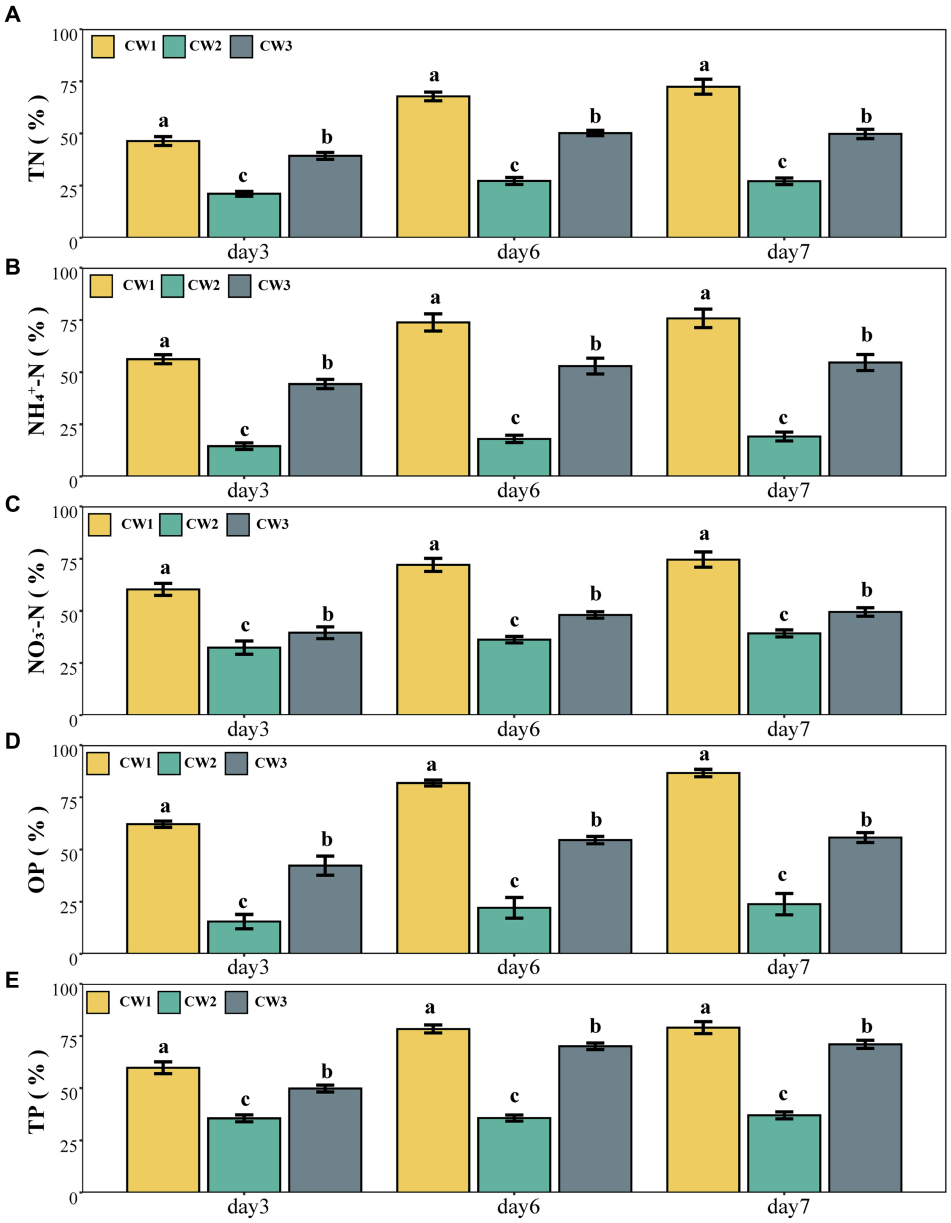
Percentage removal of **(A)** TN, **(B)** NH_4_^+^-N, **(C)** NO3^−^-N, **(D)** OP, **(E)** TP.

The analysis of variance (ANOVA) demonstrated statistically significant variations in total nitrogen (TN) levels across the effluent of all SFCW systems (*p* < 0.05). During initial week of the experiment, the wetland treatment CW1 (T1) had the highest rate of TN removal, reaching 74.81% (Figure 1). This rate surpassed the maximum performance of CW3 (51.12%) and CW2 (27.62%). The highest percentage (28.10%) of elimination attained by CW2 was observed during the period spanning from the second to the third week (Table 2).

**Table 2:** The average concentrations of effluent pollutants and their removal efficiency in each treatment wetland reactor.

Time in weeks	Parameter	Unit	Influent	CW 1		CW2		CW3	
				Effluent Conc.	R (%)	Effluent Conc.	R (%)	Effluent Conc.	R (%)
Week 1	BOD	mg/L	3,200	908.89 ± 76.88	71.6 ± 2.40	1538.3 ± 73.53	51.9 ± 2.30	1173.3 ± 50.00	63.3 ± 1.56
	COD	mg/L	7,500	1888.7 ± 240.75	74.8 ± 3.21	4892.4 ± 244.36	34.7 ± 3.26	3049.2 ± 279.39	59.3 ± 3.73
	NH_4_^+^-N	mg/L	3.85	0.78 ± 0.08	79.6 ± 2.21	3.10 ± 0.06	19.4 ± 1.54	1.61 ± 0.15	58.0 ± 3.84
	NO_3_^−^-N	mg/L	20	4.43 ± 0.66	77.8 ± 3.29	12.03 ± 0.20	39.8 ± 0.99	9.94 ± 0.36	50.2 ± 1.81
	OP	mg/L	2.93	0.36 ± 0.06	87.7 ± 1.93	2.10 ± 0.03	28.2 ± 1.15	1.28 ± 0.06	56.3 ± 2.15
	TDS	mg/L	2.000	456.78 ± 61.16	77.1 ± 3.06	1500.2 ± 23.27	24.9 ± 1.16	1047.0 ± 24.93	47.6 ± 1.25
	TN	mg/L	35	8.81 ± 1.79	74.8 ± 5.13	25.33 ± 0.34	27.6 ± 0.97	17.11 ± 0.51	51.1 ± 1.45
	TP	mg/L	12	2.27 ± 0.23	81.0 ± 1.88	7.59 ± 0.15	36.7 ± 1.29	3.35 ± 0.20	72.0 ± 1.66
	TSS	mg/L	1,800	512.67 ± 51.33	71.5 ± 2.85	676.44 ± 30.34	62.4 ± 1.69	990.67 ± 53.59	44.9 ± 2.98
	pH		7.50	6.99 ± 0.08	–	6.17 ± 0.16	–	7.45 ± 0.27	–
Week 2	BOD	mg/L	3,200	984.44 ± 109.90	69.2 ± 3.43	1556.6 ± 65.72	51.3 ± 2.05	1278.3 ± 55.00	60.0 ± 1.72
	COD	mg/L	7,500	1900.0 ± 217.89	74.6 ± 2.91	5108.6 ± 225.77	31.8 ± 3.01	3322.3 ± 316.08	55.7 ± 4.21
	NH_4_^+^-N	mg/L	3.85	0.80 ± 0.09	79.1 ± 2.37	3.10 ± 0.09	19.5 ± 2.43	1.65 ± 0.10	57.0 ± 2.67
	NO_3_^−^-N	mg/L	20	4.65 ± 0.27	76.7 ± 1.36	12.24 ± 0.37	38.7 ± 1.86	10.03 ± 0.29	49.8 ± 1.46
	OP	mg/L	2.93	0.40 ± 0.05	86.3 ± 1.65	2.11 ± 0.07	27.8 ± 2.43	1.33 ± 0.04	54.6 ± 1.51
	TDS	mg/L	2,000	503.44 ± 60.14	74.8 ± 3.01	1527.5 ± 39.06	23.6 ± 1.95	1074.6 ± 46.16	46.2 ± 2.31
	TN	mg/L	35	10.13 ± 0.47	71.0 ± 1.35	25.27 ± 0.26	27.8 ± 0.74	17.71 ± 0.32	49.4 ± 0.92
	TP	mg/L	12	2.39 ± 0.26	80.1 ± 2.20	7.39 ± 0.20	38.4 ± 1.66	3.30 ± 0.16	72.4 ± 1.32
	TSS	mg/L	1,800	557.11 ± 70.88	69.0 ± 3.94	683.11 ± 48.98	62.0 ± 2.72	966.44 ± 68.04	46.3 ± 3.78
	pH		7.50	6.87 ± 0.07	–	6.14 ± 0.08	–	7.41 ± 0.10	–
Week 3	BOD	mg/L	3,200	1096.6 ± 109.32	65.7 ± 3.42	1550.0 ± 103.11	51.5 ± 3.22	1166.6 ± 60.83	63.5 ± 1.90
	COD	mg/L	7,500	2309.6 ± 303.38	69.2 ± 4.05	5085.8 ± 362.04	32.1 ± 4.83	3709.1 ± 445.70	50.5 ± 5.94
	NH_4_^+^-N	mg/L	3.85	1.02 ± 0.10	73.4 ± 2.63	3.11 ± 0.07	19.1 ± 1.76	1.88 ± 0.05	51.1 ± 1.41
	NO_3_^−^-N	mg/L	20	5.31 ± 0.25	73.4 ± 1.23	12.18 ± 0.37	39.0 ± 1.86	9.99 ± 0.42	50.0 ± 2.08
	OP	mg/L	2.93	0.39 ± 0.03	86.7 ± 0.91	2.34 ± 0.07	20.0 ± 2.33	1.29 ± 0.07	56.0 ± 2.36
	TDS	mg/L	2,000	487.11 ± 23.85	75.6 ± 1.19	1552.7 ± 17.56	22.3 ± 0.88	1093.3 ± 51.07	45.3 ± 2.55
	TN	mg/L	35	8.87 ± 0.33	74.6 ± 0.95	25.17 ± 0.27	28.1 ± 0.78	16.93 ± 0.47	51.6 ± 1.33
	TP	mg/L	12	2.50 ± 0.10	79.1 ± 0.83	7.64 ± 0.21	36.3 ± 1.74	3.60 ± 0.12	70.0 ± 1.02
	TSS	mg/L	1,800	492.22 ± 21.99	72.6 ± 1.22	751.33 ± 144.71	58.2 ± 8.04	999.56 ± 92.51	44.4 ± 5.14
	pH		7.50	7.62 ± 0.04	–	6.02 ± 0.05	–	7.54 ± 0.05	–
Week 4	BOD	mg/L	3,200	1000.0 ± 76.49	68.7 ± 2.39	1646.6 ± 116.14	48.5 ± 3.63	1348.8 ± 151.36	57.8 ± 4.73
	COD	mg/L	7,500	2400.7 ± 251.41	67.9 ± 3.35	4608.0 ± 499.04	33.8 ± 4.78	3538.4 ± 491.39	52.8 ± 6.55
	NH_4_^+^-N	mg/L	3.85	1.12 ± 0.09	70.8 ± 2.26	3.14 ± 0.11	18.4 ± 2.79	1.84 ± 0.03	52.2 ± 0.89
	NO_3_^−^-N	mg/L	20	5.97 ± 0.29	70.1 ± 1.47	12.20 ± 0.38	39.0 ± 1.89	10.46 ± 0.39	47.7 ± 1.96
	OP	mg/L	2.93	2.57 ± 0.11	85.5 ± 1.94	2.37 ± 0.14	19.1 ± 4.67	1.29 ± 0.09	56.0 ± 3.10
	TDS	mg/L	2,000	521.89 ± 31.93	73.9 ± 1.60	1,510 ± 14.69	24.4 ± 0.73	1105.5 ± 35.13	44.7 ± 1.76
	TN	mg/L	35	10.84 ± 0.17	69.03 ± 0.49	26.32 ± 0.31	24.80 ± 0.89	18.60 ± 0.44	46.87 ± 1.26
	TP	mg/L	12	2.91 ± 0.35	75.71 ± 2.93	7.61 ± 0.15	36.61 ± 1.29	3.68 ± 0.22	69.36 ± 1.86
	TSS	mg/L	1,800	536.22 ± 38.88	70.21 ± 2.16	671.56 ± 42.28	62.69 ± 2.35	1059.11 ± 116.20	41.16 ± 6.46
	pH		7.50	7.66 ± 0.05	–	5.91 ± 0.05	–	7.59 ± 0.02	–

Table 2 presents statistically significant variations in the concentrations of NH_4_^+^-N and NO_3_^−^-N in the effluent of the three SFCW systems (*p* < 0.05). During the initial week of the experiment, T1 (79.62%) had the most significant elimination rate for NH_4_^+^-N, followed by T3 (58.07%) and T2 (19.44%) on the seventh day. During the fourth week, T3 (52.23%) exhibited a rise in NH_4_^+^-N removal as compared to 51.10% in the third week (Table 2). In the second week of treatment, T2 (19.54%) exhibited a maximum removal rate of NH_4_^+^-N, which was notably lower than T1 and T3, as depicted in Table 2.

During the second week of the experiment, T2 (38.79%) demonstrated the lowest percentage removal of NO_3_^−^-N. However, T2 exhibited satisfactory NO_3_^−^-N elimination rates compared to NH_4_^+^-N and TN. In the initial week, T2 had the highest NO_3_^−^-N removal rate, reaching 39.87%. T3 exhibited higher elimination rates for NO_3_^−^-N as compared to T2. On the seventh day of the first week, T3 reduced NO_3_^−^-N by 50.29%, which was 10.42% greater than T2. In the initial 2 weeks of the experiment, the removal percentages for T3 ranged from 50.29 to 49.84%. Subsequently, there was a slight increase, reaching 50.07% in week 3, followed by a decline to 47.71% in the last week (Table 2). T1 exhibited a remarkable NO_3_^−^-N removal efficiency of 77.84%, surpassing the removal rates observed in T2 and T3 (Figure 1).

### 3.2 Phosphorous removal

The concentrations of phosphorus and ortho-phosphorus in each SFCW effluent varied according to the hydraulic retention time, as depicted in Figure 1. There was also a significant difference in TP content among the wetland systems throughout the same operational period (*p* < 0.05). Treatment T3 (72.08%) demonstrated a significant decrease in TP concentrations within the initial week. In the first week of the experiment, T1 achieved a TP removal rate of 81.07%, surpassing T3 (72.08%) and T2 (36.79%). Conversely, the efficacy of T2 increased from 36.79 to 38.43% from the first to the second week. However, the effectiveness of T2 experienced a slight reduction during the third week, followed by a minor improvement in the fourth week, resulting in a recorded efficiency of 36.61% (Table 2).

### 3.3 BOD and COD removal

The ANOVA results indicated significant variations in BOD and COD removal among the SFCWs. For BOD, the initial efficacy of the wetland treatment T1 was 71.60% during the first week, followed by a slight decline to 69.24% in the second week. Over 3 weeks, the T2 system experienced a decrease in removal effectiveness, dropping from 51.93% in the first week to 51.56% in the third week and reaching its minimum removal efficiency of 48.54% in the fourth week (Table 2). The efficacy of T3 decreased from 63.33 to 60% over the initial and subsequent weeks, with removal efficiencies of 63.54 and 57.82% in the third and fourth weeks, respectively, indicating a notable decline (Table 2).

T1 showed efficacies of 74.82 and 74.67% for COD elimination during the first 2 weeks, respectively. The performance of T3 exhibited a persistent downward trend, with elimination percentages of 59.34, 55.70, 50.54, and 52.82% during weeks 1, 2, 3, and 4, respectively. Similar to T1, T2 also experienced a decline in performance over the initial 2 weeks, with removal efficiencies of 34.77 and 31.89%, respectively. T3 (59.70%) had the highest COD removal percentage during the first week, but its effectiveness decreased to a minimum of 50.54% in the third week, representing a decline of 9.16% compared to the first week (Table 2).

### 3.4 TDS, TSS, and pH

Significant variations in the performance of three types of modified SFCWs were observed with a hydraulic retention time of 7 days (*p* < 0.05). The wetland system T1 (77.16%) exhibited the highest removal efficiency for TDS during the initial week. However, its performance declined to 75.64% by the third week and further to 73.91% by the fourth week (Table 2). T2 achieved a peak TDS removal efficiency of 24.99%, which was 52.17% lower than T1 and 22.66% lower than T3. T3 demonstrated its highest performance in the first week with a removal efficiency of 47.65%, but this decreased to 44.72% by the fourth week (Table 2).

T1 achieved the highest efficiency for TSS removal at 72.65%, outperforming T3 by 26.34% and T2 by 9.96%. T2’s peak TSS elimination rate was 62.69% during the fourth week (Table 2). T3 showed improved TSS removal from 44.96% in the first week to 46.31% in the second week, but its performance deteriorated (41.16%) by the fourth week.

**Figure 2:**
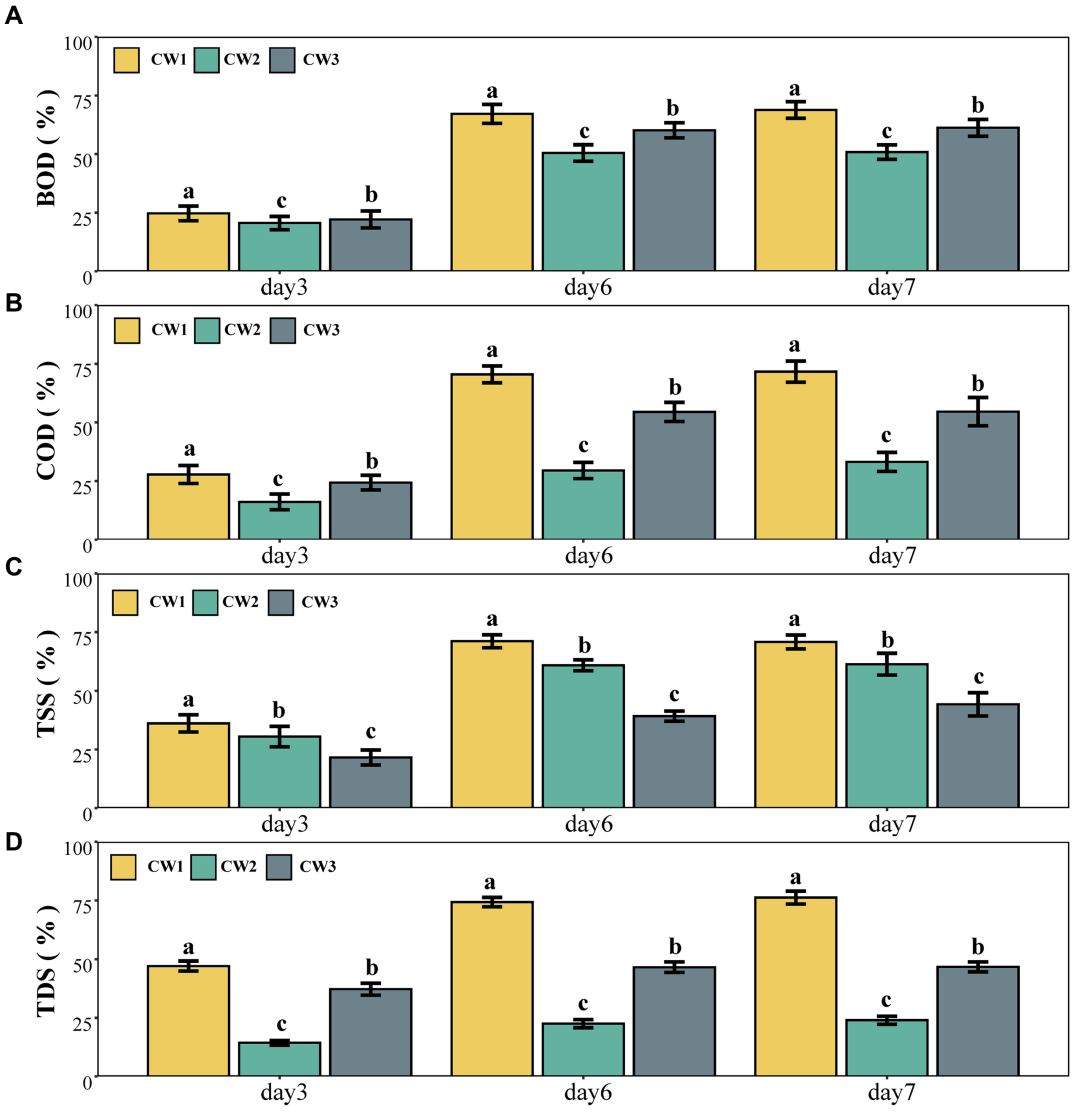
Percentage removal of **(A)** BOD, **(B)** COD, **(C)** TSS, **(D)** TDS.

Regarding pH levels, T3 recorded a higher pH (7.59) at the end of the experiment. T2 showed a continuous decline in pH, with values of 6.17, 6.14, 6.02, and 5.91 for weeks 1, 2, 3 and 4, respectively. T1 experienced a decline in pH during the first 2 weeks, followed by a consistent upward trend, reaching a peak of 7.66 by the fourth week. All three constructed wetlands exhibited a gradual decrease in pH from day 1 to day seven during the initial phase, but this decline diminished over time (Figure 2).

### 3.5 Effects of substrates and vegetation plant

Tables 3, 4 present a statistical comparison of the adsorption of specific nitrogen and phosphorus between the plants and substrates of CW1 and CW2, as well as CW1 and CW3. This comparison was conducted to assess the individual impact of these factors on the wetland reactors. The paired *t*-test was used for this analysis. A statistically significant difference (*p* < 0.05) was observed in the adsorption of NH_4_^+^-N and OP between the plants of CW1 and CW3. The adsorption of NH_4_^+^-N and OP on the substrates of CW1 and CW2 also exhibited a statistically significant difference. In contrast, it is seen that CW1 exhibits superior removal rates for NH_4_^+^-N and OP in both plants and substrates. This finding suggested that the collective utilization of plants and substrates significantly influences wastewater treatment more than their individual effects.

**Table 3:** NH_4_^+^-N and OP in the plants of CW1 and CW3.

Parameters	Day 0		Day 7		Day 14		Day 28	
	CW1	CW3	CW1	CW3	CW1	CW3	CW1	CW3
NH_4_^+^-N in plants	62.34 ± 1.17	62.34 ± 0.8	**68.41 ± 0.32**	**64.52 ± 0.31**	64.56 ± 1.14	64.56 ± 1.3	64.52 ± 1.25	64.41 ± 1.33
OP in plants	102.78 ± 1.04	102.78 ± 0.94	104.57 ± 1.42	104.53 ± 1.27	**107.21 ± 0.03**	**104.50 ± 0.05**	104.51 ± 1.10	104.42 ± 1.59

The significant statistical difference (*p* < 0.05) is indicated in bold numbers.

**Table 4:** NH_4_^+^-N and OP in the substrates of CW1 and CW2.

Parameters	Day 0		Day 7		Day 14		Day 28	
	CW1	CW2	CW1	CW2	CW1	CW2	CW1	CW2
NH_4_^+^-N in substrate	0	0	0.85 ± 0.02	0.81 ± 0.05	**0.92 ± 0.04**	**0.77 ± 0.05**	0.74 ± 0.10	0.69 ± 0.09
OP in substrate	0	0	0.88 ± 0.17	0.85 ± 0.05	**0.94 ± 0.15**	**0.79 ± 0.06**	**0.79 ± 0.02**	**0.53 ± 0.03**

The significant statistical difference (*p* < 0.05) is indicated in bold numbers.

### 3.6 Microbial community of different SFCWs

A high-throughput sequencing approach using the Illumina 16S rRNA gene was utilized to investigate the number and diversity of microbial communities in T1 and T2. The operational measures (Chao1, Shannon, and Simpson) used to assess microbial communities’ richness and diversity were identified. In July, CW1 exhibited greater richness and microbiological diversity than CW2, as evidenced by its higher Chao1, Shannon, and Simpson values. This pattern remained consistent throughout August, though there was a drop in the Chao1, Shannon, and Simpson values, as indicated in Table 5.

**Table 5:** Microbial community alpha diversity.

Month	Constructed wetland	Chao1	Shannon	Simpson
July	CW 1	1024.677 ± 266.23	7.111 ± 1.54	0.994197 ± 0.06
	CW 2	537.4286 ± 107.40	5.606602 ± 0.44	0.940525 ± 0.000364
August	CW 1	785.3163 ± 208.00	6.751978 ± 0.80	0.957896 ± 0.04
	CW 2	457.9516 ± 56.40	5.245325 ± 0.78	0.930625 ± 0.02

**Figure 3:**
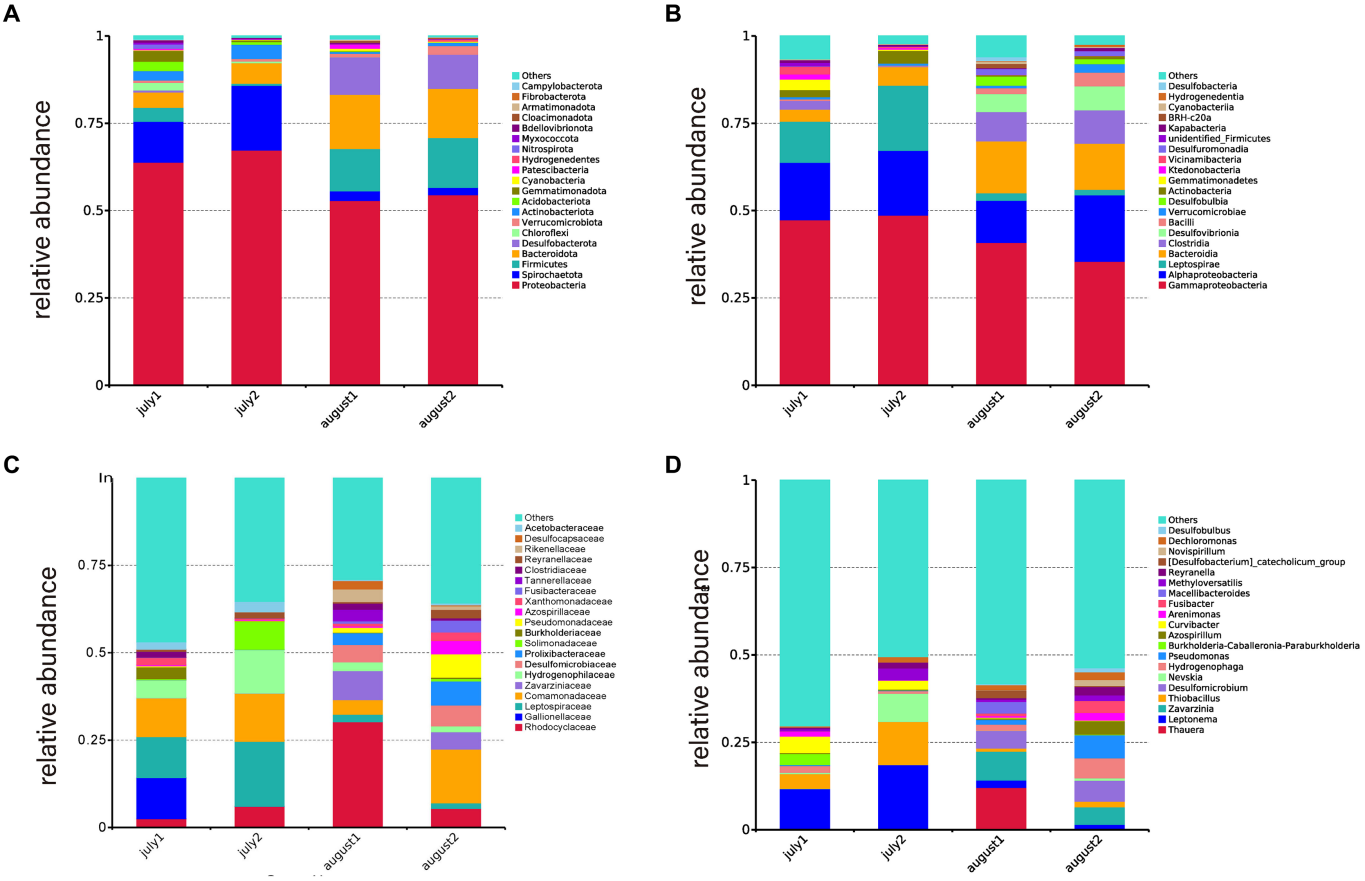
Distribution of microorganisms at different taxon levels in the different SFCW (CW1 in July: july 1, CW1 August: august 1, CW2 in July: july 2, CW2 in August: august 2), **(A)** phylum level, **(B)** class level, **(C)** family Level, **(D)** genus level.

Figure 3A illustrates the phylogenetic diversity of the microbial population at the phylum level across CW1 and CW2. During July and August, Proteobacteria were identified as the dominant taxon in both SFCW microcosms. In CW2, Proteobacteria comprised 67.30 and 54.52% of the microbial population in July and August, respectively. Similarly, in CW1, they accounted for 63.81% in July and 52.91% in August. Additionally, the substrates of both wetland systems contained other phyla such as Spirochaetota (2–18.5%), Firmicutes (0.5–14%), Bacteroidota (4.3–15.55%), Desulfobactota (0.62–10.62%), Actinobacteriota (0.45–4.06%), and Cyanobacteria (0.19–0.6%). According to Miao et al. (2015), Proteobacteria and Firmicutes are essential components in the process of denitrification. Furthermore, Bacteroidetes, Chloroflexi, Patescibacteria, Firmicutes, and Actinobacteria play crucial roles in efficiently eliminating pollutants within wetland systems. Bacteroidetes are widely recognized as polymeric organic degraders (Wang et al., 2009). Firmicutes and Chloroflexi have demonstrated significant efficacy in degrading microbial extracellular polymeric compounds and soluble microbial substances (Liu et al., 2019). Several studies indicate that Patescibacteria is a prominent bacterial phylum following the commencement of ANAMMOX and denitrification (Song et al., 2019). According to Chen et al. (2020), there is a significant correlation between the concentration of TN and gram-negative bacteria. Moreover, the matrix layer strongly correlates with denitrification, elucidating the efficient elimination of chemical oxygen demand (COD), nitrogen, and phosphorus in various wetland systems.

The microbial phylogenetic diversity at the class level in CW1 and CW2 SFCW systems is depicted in Figure 3B. The dominant groupings included Gammaproteobacteria, Alphaproteobacteria, Leptospirae, Bacteroidia, Clostridia, and Desulfovibrionia. In July, the relative abundances in CW1 were 47.35, 16.47, 11.71, 3.49, 2.47, and 0%, respectively. In CW2, their relative abundances were 48.72, 18.54, 18.59, 5.35, 0.17, and 0%, respectively. In August, the relative abundances in CW1 were 40.89, 12.03, 2.13, 14.89, 8.38, and 5.14%, respectively, while in CW2, they were 35.46, 19.03, 1.59, 13.20, 9.5, and 6.94%, respectively. According to Han et al. (2021), Proteobacteria, Alphaproteobacteria, Betaproteobacteria, and Gammaproteobacteria play significant roles in nitrification and organic matter decomposition, including anammox, nitrifying, and nitrite-oxidizing bacteria, which have important ecological roles in reducing nitrate and nitrite (He et al., 2016).

Figure 3C displays the microbial community composition at the family level. The dominant families were Rhodocyclaceae (2.48–30.25%), Gallionellaceae (11.77%), Leptospiraceae (1.59–18.59%), Comamonadaceae (4.18–15.41%), and Zavarziniaceae (0.003–8.22%). Rhodocyclaceae, a significant group of denitrifying bacteria (Lu et al., 2015), was more abundant in CW1, utilizing nitrate or nitrite as the ultimate electron acceptor and playing a role in the denitrification process (Pelissari et al., 2016). Leptospiraceae facilitate biofilm development (Song et al., 2021), while Gallionellaceae promotes nitrogen elimination (Tian et al., 2020).

Figure 3D displays the genus-level microbial community composition. The dominant genera observed were Thauera (12.09%), Leptonema (1.55–18.59%), Zavarzinia (4.96–8.22%), Thiobacillus (0.09–12.27%), and Desulfomicrobium (4.98–5.97%). Thauera, present exclusively in CW1, is recognized as a significant degrader of aromatic compounds in wastewater (Mao et al., 2010) and plays a crucial role in removing nitrogen and phosphorus from low-carbon source sewage (Ren et al., 2021). Leptonema, a member of the Leptospiraceae family, plays a crucial role in the production of lipopolysaccharides and facilitates biofilm development (Song et al., 2021). Zavarzinia, an Alphaproteobacteria, can use carbon monoxide as a source of energy and is primarily responsible for breaking down benzene in oil-sands-contaminated water (Rochman et al., 2017; Lee et al., 2019). Thiobacillus converts NO_3_^−^-N and NO_2_^−^-N into N_2_ under facultative anaerobic conditions (Huang et al., 2018) and is categorized as a sulfide-oxidizing bacteria (SOB) in constructed wetlands (Samsó and García, 2013).

**Figure 4:**
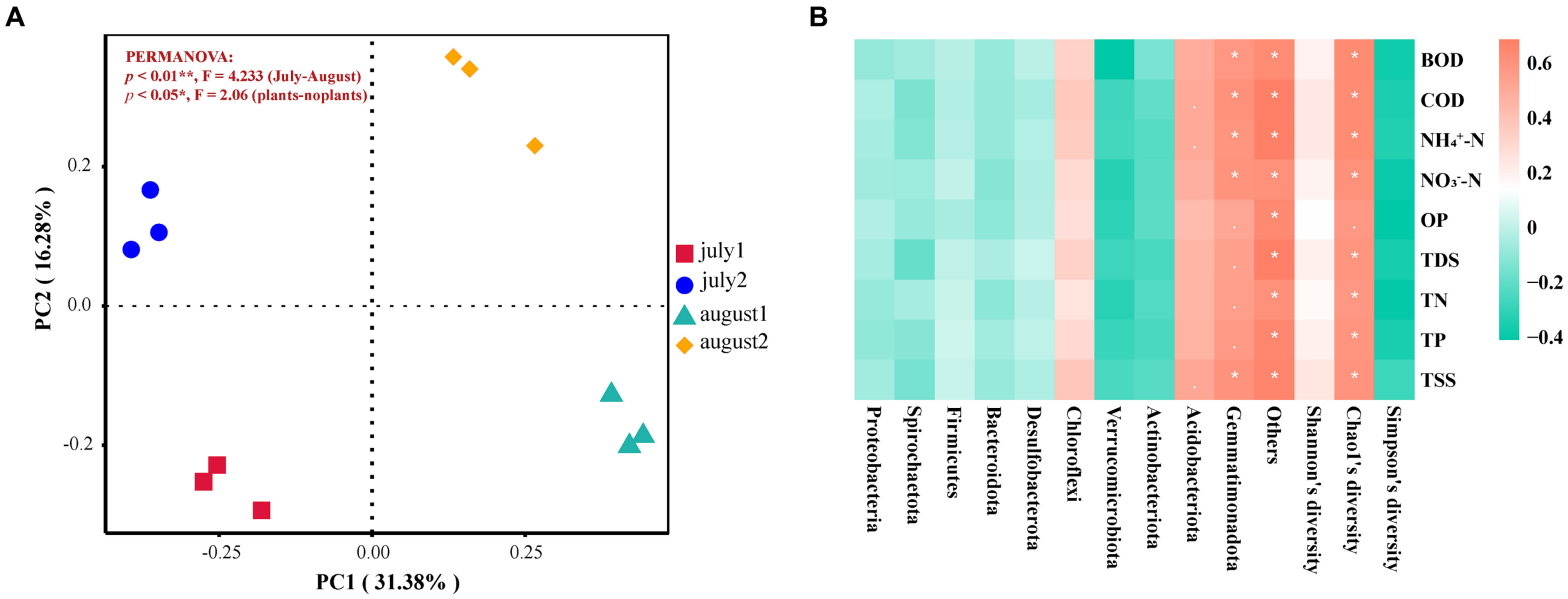
**(A)** PCoA Analysis of microbial community. july 1: at the start of the experiment with plant; july 2: at the start of the experiment without plant; august 1: at the end of the experiment with plant; august 2: at the end of the experiment without plant, **(B)** Correlation Heat Map Between Microbial Community Composition and Pollutant Removal Efficiency.

Principal Coordinate Analysis (PCoA) is a valuable method for visually representing the dissimilarities or similarities between several groupings. The findings indicate that PC1 and PC2 accounted for 31.38 and 16.28% of the contribution rates, respectively (Figure 4A). In July, the proximity of the bacterial communities in the substrates of CW1 and CW2 suggests a similarity in their community makeup. Conversely, bacterial communities exhibited more dispersion in August, indicating significant differences in their composition and poor resemblance across the communities. The comparison reveals notable disparities in the distribution distances of communities, suggesting substantial dissimilarities in the makeup of CW1 and CW2.

Figure 4B illustrates the correlation between the microbial community and various pollutants, including BOD, COD, NH_4_^+^-N, NO_3_^−^-N, OP, TDS, TN, TP, and TSS. Chloroflexi exhibits a strong positive correlation with the removal of BOD and TN, indicating its significant role in eliminating these pollutants. Acidobacteriota is positively associated with BOD and NH_4_^+^-N and shows a strong correlation with TN. Gemmatimonadota also demonstrates significant positive relationships with various parameters, including TN, OP, and BOD. Additionally, the Chao1 index exhibited a strong positive correlation with several pollutants, such as BOD, COD, NH_4_^+^-N, NO_3_^−^-N, TDS, and TN, suggesting that greater microbial diversity effectively enhances the removal of these pollutants.

## 4 Discussion

### 4.1 Total nitrogen, NH_4_^+^-N and NO_3_^−^-N removal

*Lemna minor* L. effectively adsorbed nitrogen from wastewater in the wetland system, yielding better results within a short period of 7 days, especially when substrates such as lignite and brick rubble were present. Nitrification and denitrification are the primary processes influencing the movement and transformation of nitrogen, carried out mainly by nitrifying and denitrifying bacteria (Fan et al., 2013; Sani et al., 2013). However, the denitrification process in constructed wetlands, responsible for removing total nitrogen (TN), may be adversely influenced by several factors (Zhou et al., 2018). To overcome these constraints, lignite can serve as an effective carbon source and exhibit the system’s adsorption characteristics while avoiding pollution (Zhou et al., 2017). Brick rubble, with its porous structure, creates anaerobic conditions and provides a large surface area to promote the growth of biofilms rich in denitrifying bacteria, thereby reducing TN (Lima et al., 2018; Li H. et al., 2021). CW1, comprising substrates and plants, demonstrated a significantly higher capacity (74.8%) for total nitrogen removal from wastewater than CW2 and CW3. *Lemna minor* L. has been found to have a high capacity for nitrogen removal (Kern and Idler, 1999; Patel and Kanungo, 2010). Cedergreen and Madsen (2002) reported that *Lemna minor* L. can uptake NH_4_^+^-N and NO_3_^−^-N from water, with both roots and leaves absorbing nitrogen from wastewater, enhancing nitrogen removal capabilities. Substrates also play a crucial role in eliminating pollutants from wastewater. Findings from the CW2 study (Table 4), which focused exclusively on substrates, indicate that bricks can adsorb nitrogen from wastewater. Ren et al. (2007) demonstrated the effectiveness of using bricks as a substrate in wetlands for removing nitrogen and phosphorus from wastewater. An increase in the hydraulic retention time had improved the efficiency of all wetlands. The duckweed plant significantly contributed to this process, showing that longer hydraulic retention times led to higher removal rates of contaminants (Jing et al., 2002; Adhikari et al., 2015).

### 4.2 Phosphorous removal

The results from CW3 (Table 3) demonstrate that *Lemna minor* L. can adsorb phosphorus. Furthermore, its adsorption capacities are improved in the presence of substrates, as shown by CW1. CW1 exhibited better phosphorus removal than CW2 and CW3 (Table 2). According to a study by Perniel et al. (1998), *Lemna minor* L. monoculture consistently exhibited the highest phosphorus removal capacity from stormwater within 8 weeks. In the current study, *Lemna minor* L. utilized phosphate as a growth substance, resulting in a substantial decrease of 81% in total phosphorus levels in CW1 over 7 days. This reduction can be attributed to absorption, adsorption, or direct uptake by the plant. Iqbal et al. (2019) examined *Lemna minor* L. (duckweed) growth and nutrient removal efficiency from synthetic and dumpsite leachate in both artificial and natural environments, finding that *Lemna minor* L. effectively removes nutrients in both settings. Dinh et al. (2020) employed a laboratory-scale stabilization pond to cultivate duckweed using anaerobically treated wastewater diluted by a factor of 10, achieving 85% TP removal within 5 days. Ceschin et al. (2019) identified duckweed as a viable plant species for wastewater bioremediation due to its ability to withstand and absorb a wide range of contaminants and significant amounts of nutrients. Duckweed exhibits continuous vegetative development throughout the year, achieving high rates by utilizing nutrients from wastewater, which can accumulate within the cells or be used to generate new biomass.

Bojcevska and Tonderski (2007) operated surface flow constructed wetlands planted with *Cyperus papyrus* and *Echinochloa pyramidalis* for 12 months, observing 48–84% TP removal. In the present experiment, comparable TP removal was observed in CW1 within 7 days, indicating effective results in a short time. The substrate media plays a crucial role in phosphorus removal (Vymazal, 2011). In our experiment, CW1 removed more phosphorus than CW3, likely due to the presence of substrates. Bricks, being porous media, have an increased surface area when crushed, aiding in phosphorus adsorption from wastewater (Li J. et al., 2021). Constructed wetlands mainly get oxygen from atmospheric diffusion and plant roots (Zhou et al., 2019). Bricks as substrates may be responsible for the observed phosphorus reduction in CW2. They exhibit qualities to effectively remove phosphorus from wastewater, making them a suitable substrate (Ren et al., 2007).

The substrates in CW2 demonstrated the capacity to remove phosphorus from the influent effectively (Table 4). Additionally, these substrates can facilitate the proliferation of microbial communities and plants. The combined utilization of plants and substrates resulted in a greater phosphorus removal efficiency of 81% for CW1 (Table 2) compared to CW2 and CW3. A study by Wang et al. (2013) assessed the physicochemical characteristics and phosphorus adsorption capabilities of oyster shells, broken bricks, volcanic rock, and zeolite as substrates for treating swine wastewater. All substrates, except volcanic rock, demonstrated suitability for enhancing microorganism and plant development in water treatment systems. Billore et al. (2001) treated distillery effluent in a constructed wetland consisting of four cells. The effluent from cells one and two was directed to cells three and four, which contained plants and brick debris. Following a pretreatment process, the effluent decreased 79% in phosphorus content. Shi et al. (2017) documented a 29.16% reduction in phosphorus using red bricks in a wetland system.

### 4.3 BOD and COD removal

*Lemna minor* L. has demonstrated potential for effectively removing BOD. The combined effects of plants and substrates, particularly the porous structure of bricks, enhanced the pollutant removal efficiency. The present experimental findings indicate that plants effectively removed a significant proportion of BOD and COD in CW3 (Table 2). The combined influence of plants and substrates in CW1 significantly impacts the removal of BOD and COD, resulting in notable reductions in these pollutants (Table 2). Körner et al. (2003) conducted a study examining the efficacy of duckweed in treating wastewater. Their findings indicated that duckweed facilitated the breakdown of organic matter, in terms of BOD and COD, by providing increased oxygen and a larger surface area for bacterial proliferation. Duckweed achieved a removal rate of 67.4% for COD and 95.8% for BOD after 20 days in a study by Oron et al. (1987). However, in the current experiment, *Lemna minor* L. removed 63.3% of BOD and 59.3% of COD within 7 days. Mandi (1994) conducted an experiment treating water with the duckweed species *Lemna gibba* at low organic loading, leading to an 82% reduction in wastewater’s COD. Adhikari et al. (2015) found similar results when they introduced a mixture of diluted raw dairy manure into a combination of surface flow and subsurface flow wetlands using duckweed as vegetation. The removal of COD in primary duckweed wetlands ranged from 3 to 81%, whereas in secondary duckweed wetlands, it varied from 35 to 38%. These findings are consistent with the results obtained in the current experiment. The BOD of wetland systems CW1 and CW3 abruptly increased on the sixth day due to the generation of gaseous oxygen resulting from the photosynthetic processes of *Lemna minor* L.

The CW2 substrates demonstrated a significant reduction in BOD and COD, indicating their ability to adsorb these pollutants from wastewater, as shown in Tables 2, 4. Saeed et al. (2018) conducted an experiment demonstrating a noteworthy reduction of 83.2% in COD and 87.0% in BOD through the utilization of a hybrid-built wetland system with bricks as the substrate medium. These findings coincide with the outcomes of the present study, which showed that the surface flow-constructed wetland with brick rubble and *Lemna minor* L. has the potential to remove BOD and COD from wastewater.

### 4.4 TDS, TSS, and pH

*Lemna minor* L. was primarily responsible for the removal of TDS from the wetland systems. The elimination of TDS was more significant in CW1 than in CW2 and CW3, with CW3 showing a higher reduction than CW2, as indicated in Table 2. The elevated total dissolved solids (TDS) removal can be attributed to the presence of *Lemna minor* L. A study by Amare et al. (2018) demonstrated a 68% reduction in TDS using *Lemna minor* L. in wastewater treatment in tropical semiarid areas of Ethiopia. Ali et al. (2024) constructed a free-water surface flow wetland planted with *Pistia stratiotes* and observed significant removal of TDS and TSS with a hydraulic retention time (HRT) of 30 days. In our experiment, a comparable amount of TDS and TSS removal was observed with an HRT of just 7 days, indicating that CW1 is capable of efficiently removing TDS and TSS in a short period. The substrates were shown to be responsible for the higher clearance rates of TSS seen in CW1. Findings from CW2 validate; bricks (due to their expansive surface area and pores) functioned as filters and effectively absorbed total suspended particles from wastewater. A study by Javani et al. (2016) produced similar results, showing that geotextile sheets and structural brick debris significantly impacted the removal of TSS contaminants in treated municipal wastewater. Saeed et al. (2018) conducted an experiment using recycled bricks to treat industrial wastewater, with findings consistent with our experiment, indicating that bricks are valuable for removing TSS. The present experiment revealed that the elimination of TSS in CW2 was significantly higher than in CW3, attributed to the porous structure of the bricks. Obeng et al. (2023) also found that clay bricks effectively eliminated TSS from wastewater. While bricks are crucial for eliminating TSS, the findings from CW3 suggest that *Lemna minor* L. can also remove TSS from wastewater. Consistent with our results, Papadopoulos and Tsihrintzis (2011) reported a 63% reduction in TSS using *Lemna minor* L. in wastewater treatment. As indicated in Table 2, a pH decrease was noted at the start of the experiment, particularly in CW2. Throughout the experiment, the continuous pH decrease in CW2 is linked to the presence of lignite, known for its pH-lowering effect due to its limited negative charge, reducing the removal action of H+ ions (Di et al., 2022). The pH value from CW3 provides additional evidence supporting the pH decline observed in CW1 and CW2, attributed to the presence of lignite. However, the pH levels showed a noticeable upward trend during the second week. The observed increase in the ultimate pH values of 7.66 for CW1, 7.37 for CW2, and 7.59 for CW3 can be linked to the photosynthetic process in the plant.

### 4.5 Microbial community

Brick rubble created an optimal environment for the growth of the microbial community due to its extended surface area and porous structure. Chloroflexi, Acidobacteriota, and Gemmatimonadota were identified as key contributors to the removal of BOD, COD, nitrogen, and phosphorus. In the present experiment, Proteobacteria exhibited the highest abundance at the phylum level. Additionally, at the class level, α-Proteobacteria and γ-Proteobacteria were identified as the most abundant microorganisms (Figure 3). These microorganisms play a crucial role in the degradation of organic matter and nitrification processes and are commonly encountered in sewage treatment systems (Chen et al., 2019; Al Ali et al., 2020). Table 5 demonstrates an increase in the variety and abundance of microbial communities on the substrates of CW1. The increased microbial diversity can be attributed to the presence of brick rubble, which possesses porous qualities that promote microbial development. Shi et al. (2017) reported a comparable microbial composition in a constructed wetland containing diverse construction wastes, such as clay bricks. The predominant phylum identified in their study was Proteobacteria. The purification effects of recycled aggregates derived from construction waste as fillers in created wetlands were investigated by Li et al. (2022). They observed that red bricks exhibited the highest efficiency in terms of microbial community richness. The most prevalent microbial phylum identified was Proteobacteria (Li et al., 2022), which aligns with the findings of the current experiment. Wang et al. (2020) observed that members of the Chloroflexi phylum play a crucial role in nitrogen removal. Similarly, Huber et al. (2022) demonstrated that Acidobacteriota not only remove nitrogen but also phosphorus from wastewater and act as organic carbon degraders. Mujakić et al. (2023) reported that Gemmatimonadota is involved in the removal of various pollutants, including nitrogen and phosphorus. These findings align with our experimental results, where Chloroflexi, Acidobacteriota, and Gemmatimonadota contributed to the removal of nitrogen, phosphorus, biochemical oxygen demand, and chemical oxygen demand (Figure 4B).

The observed variations in microbial communities in the PCoA analysis (Figure 4A) can be associated with the presence of vegetation. The substrates of CW1, planted with *Lemna minor* L., exhibited a higher richness of microbial communities compared to CW2, which did not have any vegetation. Menon et al. (2013) reported similar results, indicating that variations in the bacterial population of the sediment were linked to the specific plant regime employed in their study. Wang et al. (2016) found that the presence of plants positively impacted both the abundance and diversity of microorganisms in a subsurface flow-constructed wetland. These findings align with the results obtained in the current experiment. Therefore, the presence or absence of plant species significantly influenced the composition of the microbial community in the constructed wetland system (Zhang et al., 2010).

Adding brick rubble, lignite, and *Lemna minor* L. to CW1 resulted in distinct bacterial communities at the phylum, class, family, and genus levels. The brick rubble and lignite dissolved organic compounds from wastewater due to their porous structure, promoting microbial growth. The substrates, along with the vegetation plant *Lemna minor* L., supported a rich and diverse microbial community for pollutant degradation. It partially explains the high pollutant removal rates of the SFCW wetland systems.

## 5 Conclusion

This research examined the composition of the microbial community and the effects of different wetland configurations on the purification of artificial wastewater generated by the sugar industry. *Lemna minor* L. plants assimilated nitrogen, phosphate, and other contaminants. Lignite functioned as a carbon source, promoting the growth of both plants and microbial life in the substrates. These substrates served as efficient filter media, with their porous structure providing adequate space for a thriving microbial population. Plants regulated the diversity of the microbial population. The findings clearly demonstrate that *Lemna minor* L. and brick rubble have significant potential for efficiently removing nutrients from wastewater, particularly sugar mill effluent. Our results support the hypothesis that a constructed wetland system utilizing plants and substrates is effective in treating effluents from sugar industries. This effectiveness is attributed to the rich microbial community fostered by the substrates and plants within the system. This study aims to improve the removal of contaminants from wastewater treatment plants, specifically those connected to sugar mills, by utilizing economical and widely available materials. Additionally, it contributes to the global goal of achieving carbon neutrality and can be easily implemented in underdeveloped nations worldwide.

## Data availability statement

The sequencing data that support the findings of this study are available in SRA database at https://www.ncbi.nlm.nih.gov/sra/PRJNA1132581, accession number PRJNA1132581.

## Author contributions

HA: Data curation, Visualization, Writing – original draft. YM: Data curation, Formal analysis, Methodology, Writing – review & editing. XY: Funding acquisition, Project administration, Supervision, Writing – review & editing. YK: Writing – review & editing, Conceptualization, Supervision. PM: Data curation, Writing – review & editing. SA: Data curation, Writing – review & editing.

## Footnotes

^1^ https://github.com/usadellab/Trimmomatic

^2^ https://cutadapt.readthedocs.org/

^3^ https://github.com/QIIME2/q2-feature-classifier

^4^ http://www.arb-silva.de/
