# Supplementary material for: Composition of the microbial community in surface flow-constructed wetlands for wastewater treatment
Source: Front Microbiol. 2024 Jul 19;15:1421094. doi: 10.3389/fmicb.2024.1421094 (PMC11296210; doi:10.3389/fmicb.2024.1421094)
Supplement: Supplementary file 1 [file Table_1.DOCX]

Supplementary Material

**Composition of the microbial community in surface flow-constructed wetlands for wastewater treatment**

**Haider Ali^1,2^, Yongen Min^1,2^, Xiaofei Yu^1,2,3*^, Yahya Kooch^4*^, Phyoe Marnn^1,2^, Sarfraz Ahmed^5^**

^1^Engineering Research Center of Low-Carbon Treatment and Green Development of Polluted water in Northeast China, Ministry of Education & State Environmental Protection Key Laboratory For Wetland Conservation and Vegetation Restoration, School of Environment, Northeast Normal University, Changchun, China

^2^Key Laboratory of Vegetation Ecology of Ministry of Education & Key Laboratory of Geographical Processes and Ecological Security of Changbai Mountains, Ministry of Education, School of Geographical Sciences, Northeast Normal University, Changchun, China

^3^Heilongjiang Xingkai Lake Wetland Ecosystem National Observation and Research Station & Key Laboratory of Wetland Ecology and Environment & Jilin Provincial Joint Key Laboratory of Changbai Mountain Wetland and Ecology, Northeast Institute of Geography and Agroecology, Chinese Academy of Sciences, Changchun, China

^4^Faculty of Natural Resources & Marine Sciences, Tarbiat Modares University, Noor, Iran

^5^ School of Life Sciences, Northeast Normal University, Changchun, China

*** Correspondence:**
Xiaofei Yu
[yuxf888@nenu.edu.cn](mailto:yuxf888@nenu.edu.cn)

Yahya Kooch
[yahya.kooch@modares.ac.ir](mailto:yahya.kooch@modares.ac.ir)


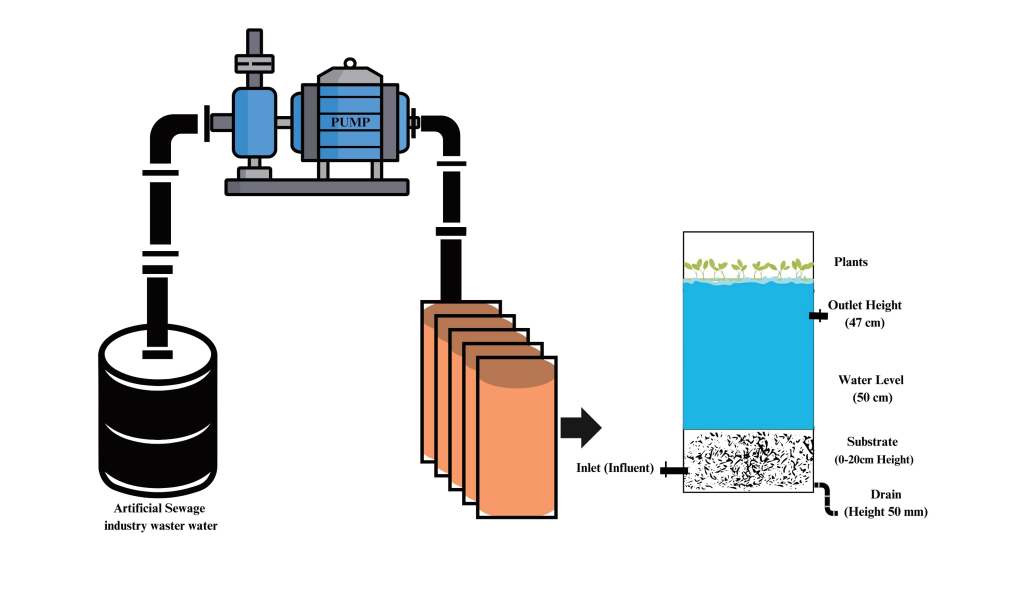


**Fig S1.** Experimental setup of SFCWs with modified substrates
